# Four-dimensional intracardiac echocardiography for guidance of tricuspid transcatheter edge-to-edge repair: a case report

**DOI:** 10.1093/ehjcr/ytae489

**Published:** 2024-09-24

**Authors:** Mark M P van den Dorpel, Claire Ben Ren, Nicolas M Van Mieghem

**Affiliations:** Department of Cardiology, Cardiovascular Institute, Thoraxcenter, Erasmus University Medical Center, office Nt-645, Dr. Molewaterplein 40, 3015 GD Rotterdam, The Netherlands; Department of Cardiology, Cardiovascular Institute, Thoraxcenter, Erasmus University Medical Center, office Nt-645, Dr. Molewaterplein 40, 3015 GD Rotterdam, The Netherlands; Department of Cardiology, Cardiovascular Institute, Thoraxcenter, Erasmus University Medical Center, office Nt-645, Dr. Molewaterplein 40, 3015 GD Rotterdam, The Netherlands

**Figure:**
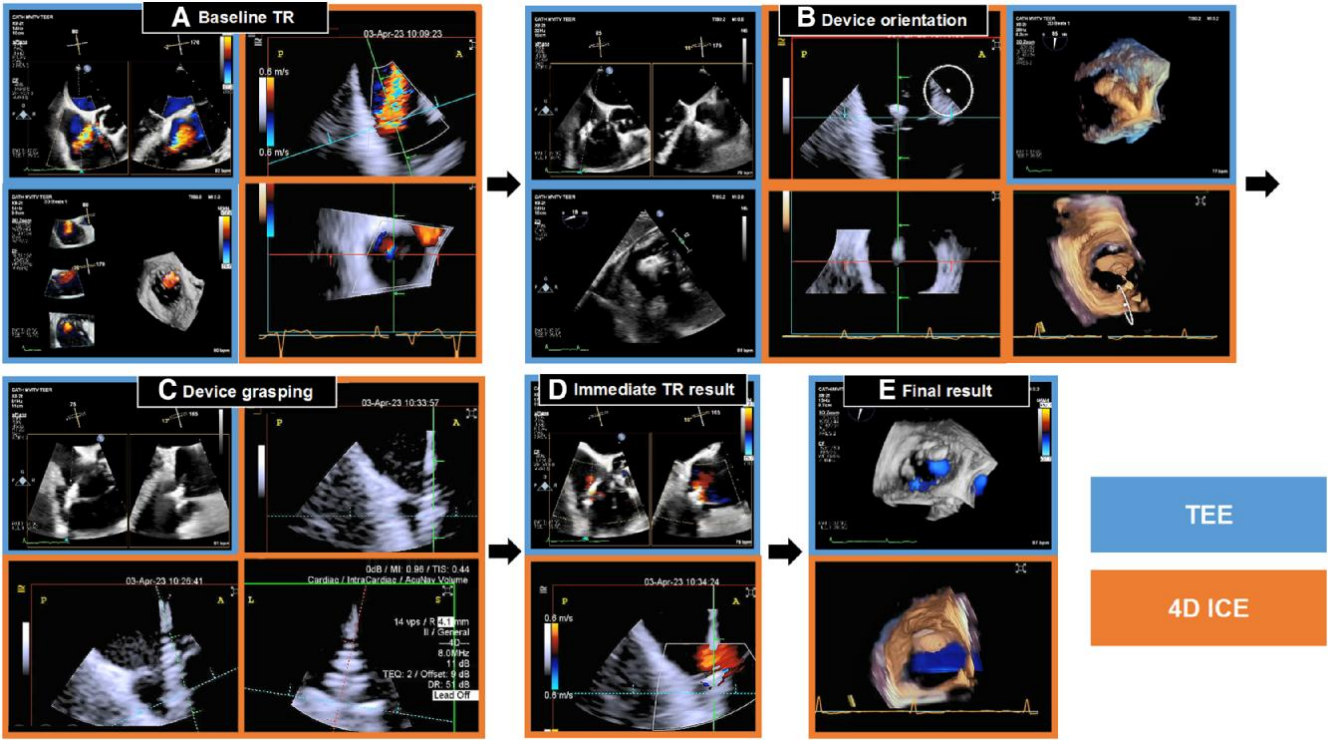


Transoesophageal echocardiography (TEE) is considered the standard imaging guidance tool for tricuspid transcatheter edge-to-edge repair (TEER). Common limitations of TEE imaging of the tricuspid valve (TV) include lower far-field spatial resolution and acoustic shadows stemming from calcific structures and metal containing intracardiac devices. The tricuspid leaflets may be particularly challenging to visualize during tricuspid TEER procedures. Intracardiac echocardiography (ICE) may overcome these intrinsic limitations.

A novel ICE catheter (ACUSON AcuNav Volume 4D ICE Catheter, Siemens, Erlangen, Germany) allows real-time 4D acquisition of high-quality 2D and 3D images, as well as simultaneous multi-planar reconstructions of the 3D data, like conventional 3D TEE.

We compared 4D ICE and TEE imaging during a tricuspid TEER procedure (*Figure 1*; Supplementary material online, *Video S1*). All key TEE imaging views for tricuspid TEER in terms of device orientation, leaflet capturing, and tricuspid regurgitation assessment could be obtained with 4D ICE (*Panels A–E*). Particularly, 4D ICE could generate the imaging view where the TEER device perpendicularity (to the leaflet coaptation line) can be assessed, like the TEE transgastric TV en-face view (*Panel B*).

In our case, 4D ICE image quality was at best equivalent to that of TEE. Thus, we currently consider 4D ICE as complementary to TEE. It may be considered as an alternative to TEE in cases in which TEE is not feasible or fails to accurately visualize the TV.

Key imaging views for tricuspid transcatheter edge-to-edge repair were obtained by transoesophageal echocardiography and intracardiac echocardiography. 4D ICE, 4-dimensional intracardiac echocardiography; TEE, transoesophageal echocardiography; TR, tricuspid regurgitation.

## Supplementary Material

ytae489_Supplementary_Data

## Data Availability

The data underlying this article will be shared on reasonable request to the corresponding author.

## Supplementary material

**Consent:** The authors confirm that written consent for submission and publication of this case report including images and associate text has been obtained from the patient in line with the Committee on Publication Ethics (COPE) guidance.

**Funding:** None declared.

